# Changing Pattern of Childhood Celiac Disease Epidemiology: Contributing Factors

**DOI:** 10.3389/fped.2019.00357

**Published:** 2019-08-29

**Authors:** Alina Popp, Markku Mäki

**Affiliations:** ^1^Faculty of Medicine and Health Technology, Tampere Center of Child Health Research, Tampere University and Tampere University Hospital, Tampere, Finland; ^2^National Institute for Mother and Child Health “Alessandrescu-Rusescu”, University of Medicine and Pharmacy “Carol Davila”, Bucharest, Romania

**Keywords:** celiac disease, changing pattern, incidence, prevalence, infant feeding, autoimmunity, awareness, screening

## Abstract

Up until the 1960s and 1970s, diarrhea, malabsorption syndrome, and failure to thrive were the presenting symptoms and signs of celiac disease (CD) in young infants; however this disease was also at the same time reported to be disappearing. Indeed, clinical childhood CD was seen to transform into a milder form, resulting in an upward shift in age at diagnosis during the 1970s (and years later for many countries). This changing pattern of CD presentation then altered the epidemiology of the disease, with major differences between and within countries observed. An awareness of the changing clinical nature of CD and use of case-finding tools to detect even clinically silent CD became an important factor in this changing epidemiology. Countries report both low and high prevalence but it seems to be on the increase resulting in a population-based level of 1–2%. This paper discusses the potential causes and environmental factors behind these observed clinical changes, identifying new clues from different studies published at the time this transformation took place. For instance, it was found that breastfeeding postponed the diagnosis of the disease but did not altogether prevent it. Moreover, gluten introduction at a young age, specifically at the mean age of 2 months, seemed to also have a clear impact in inducing malabsorption syndrome and failure to thrive in young infants in addition to other factors such as gluten intake volume and type of cereal present in the weaning food. Further, the impact of cow's milk and its high osmolarity might have played an important role; humanized milk formulas were not yet invented. Future epidemiological studies on the contributing environmental factors to the shift in CD presentation are thus recommended for countries in which these changing clinical features are still being observed.

## Introduction

Celiac disease (CD) has earlier been considered to be a rare intestinal disease occurring only in children, a disease that Samuel Gee in 1888 presented as the “coeliac affection” (1). As summarized by John Walker-Smith, Gee was very accurate in his description of childhood CD: “it is a kind of chronic indigestion, which is especially apt to affect children between 1 and 5 years old and where signs of the disease are yielded by the feces, being loose, but not watery, bulky, and pale. The onset of the disease is usually gradual and cachexia is a constant symptom. The belly is mostly soft and often distended” (2). The conception on CD changed in early 1950s by the discoveries of Dicke, who showed that the cause of harm in patients with CD was dietary gluten; in fact, he specifically noted that the disease was not caused by all cereals but, specifically, wheat flour (3). The next major research discovery occurred in the 1950s and involved the use of a peroral intestinal suction biopsy apparatus as a diagnostic tool. When gluten was ingested by children with CD, the development of a characteristic mucosal lesion in the jejunum was observed, namely villous atrophy with crypt hyperplasia (4). Moreover, new biopsy criteria were adopted early on in Europe by the European Society for Pediatric Gastroenterology, even for young infants (4–6).

Celiac disease (CD) was long thought to be a rare disease, occurring only in children with a classical presentation known as malabsorption syndrome and failure to thrive (7). However, today, CD is known as an autoimmune systemic disorder occurring in genetically susceptible individuals and perpetuated by the daily ingestion of gluten cereals (i.e., wheat, rye, and barley) with manifestations both in the small intestine and in extraintestinal organs (7–10). CD is more complex than simple intestinal malabsorption, which is, in fact, no longer essential for diagnosis. Another important milestone in CD diagnostics and screening was the discovery and clinical use of highly CD-pathognomonic, circulating, gluten-dependent tissue autoantibodies measured within the immunoglobulin A (IgA) class (11–18). Awareness of the disease and use of these tools have come to determine the epidemiological outcome in CD research. Furthermore, late-developing, small-intestinal mucosal injury also plays a role in the changing pattern and epidemiology of CD. In other words, this refers to a CD latency in which the disease exists but is not manifested at the mucosal level, even if the patient has ingested gluten for decades (9). Susceptibility to CD is inherited; however, upon a child's first encounter with gluten he may or may not develop the disease at a small-intestinal mucosal level soon thereafter. Oral tolerance toward gluten can persist for a long period of time with the deterioration of the mucosa possibly taking place at a later age. This also explains the late mucosal relapses during gluten challenges and late appearing mucosal injury concordance in monozygotic twins as well as possibly spontaneous mucosal recoveries upon continued gluten ingestion.

Celiac disease (CD), known as a rare malabsorption syndrome in young Caucasian children has, since the 1970s, come to be known as a common, chronic, food-induced autoimmune systemic disorder world-wide, diagnosed in children, adolescents, adults, and the elderly. The changes in perception of CD as a clinical disease and observed changes in occurrence have been remarkable during the past 50 years. In these aspects, there are, however, several differences between countries and continents. This review thus discusses the pattern of change in childhood CD presentation, its influence on its epidemiology, increasing incidence rates, and the causes behind them.

## Changing Clinical Features

**Figure 1:**
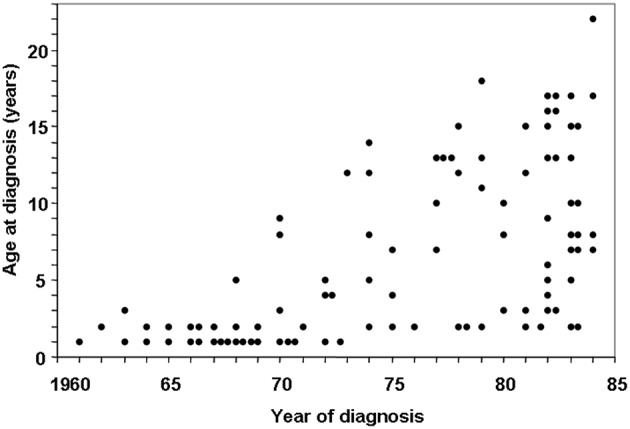
Changing pattern of childhood celiac disease affecting epidemiology results in Finland in 1960–1984. Decreasing numbers of cases were diagnosed in infancy and increasing numbers diagnosed at school age and adolescence after 1972. [Reproduced with permission from Mäki et al. (23)].

In the 1960s, a child with CD typically presented with prolonged diarrhea, malabsorption syndrome, and failure to thrive, often before the age of 2 (7). However, a shift in CD presentation became evident during the 1970s and 1980s, with childhood CD reportedly disappearing altogether (19–22). In Finland, however, from 1970 onward, it was observed that, in addition to the disappearance of the classical form of CD in young children, the incidence rates of the milder forms of the disease increased, resulting in an upward shift in age at diagnosis from 2 to 8 years (Figure 1) (23). This was also evident in the UK, with a changing disease pattern seeming to be a fact; childhood CD tended to present at later age (24).

**Figure 2:**
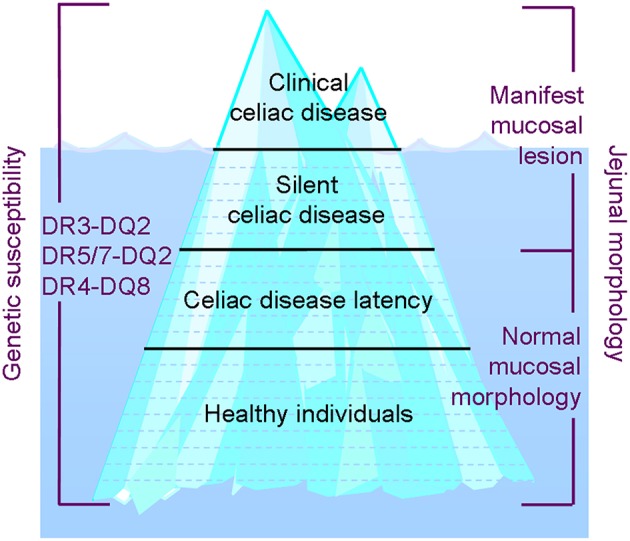
The celiac disease (CD) iceberg and the spectrum of celiac trait: Awareness of different clinical forms of CD and clinicians' activity in case-finding by screening will influence the outcome of incidence rates and prevalence in different countries and within countries.

When CD manifests during the later stages of childhood, symptoms can be gastrointestinal; however the disease is often monosymptomatic, presenting with short stature, delayed puberty, and joint pain or anemia due to isolated iron deficiency (23, 25–30). Gastrointestinal symptoms may also be mild or non-existent. If the healthcare professionals are diagnosing only classical CD presenting at young age and older children with gastrointestinal symptoms, only the tip of the iceberg of all CD patients are detected (Figure 2), epidemiologically resulting in low incidence and prevalence numbers. These clinically silent patients, i.e., children with no symptoms or signs suggesting CD, also exhibit a gluten-triggered and -dependent small intestinal mucosal lesion manifestation (Figure 2). They require an increased awareness of healthcare professionals and a case-finding strategy for early diagnosis. Specifically, celiac autoantibody testing can determine an underlying gluten-induced disease, yielding high sensitivity and specificity (11–18). The existence of clinically silent patients became evident when performing gluten challenges to earlier diagnosed well-treated patients (31, 32) in addition to CD screening for certain risk groups, such as those with type 1 diabetes (13, 18) or healthy, first-degree family members (33, 34). Case-finding using celiac-pathognomonic serum IgA class R1-type reticulin autoantibodies (11, 12), later known as endomysial and tissue transglutaminase antibodies (14, 15), detected the clinically silent patients and helped change the perception of childhood CD. The changing pattern of CD epidemiology would partly go unnoticed, if healthcare professionals are not aware of the existence of clinically silent CD and use a case-finding strategy in diagnostics.

Latent CD refers to the fact that the disease exists but does not manifest at the mucosal level until at some later age. The small intestine mucosa can stay intact upon years and decades of full gluten ingestion, or the mucosal damage can develop gradually. Final deterioration of the mucosa occurs when oral tolerance toward gluten ceases (9, 35) (Figure 2). We have extensively discussed latent and developing CD in a recent review article (9). Latent CD patients may be serum autoantibody positive, have a normal small intestine mucosa and thus according to biopsy criteria be excluded for CD. These patients may even become serum autoantibody negative upon normal gluten ingestion but still develop the disease at some later age (36). Stenhammar et al. addressed the latency issue in CD family studies spanning 20 years (37, 38). When a small intestine biopsy was performed for 100 first-degree relatives of index patients with childhood CD in 1982, only 2% were found to have clinically silent CD. When they reinvestigated all first-degree relatives of the same index patients 20–25 years later, eight new cases of CD were detected (8.3%), two of whom were found not to have the disease in the first study. Eleven more relatives exhibiting mild enteropathy were identified in the new screening, and it was suggested that they were followed carefully for potential development to overt disease. CD incidence and prevalence figures are influenced by the age of the patients at time of study and also whether a changing pattern of childhood CD has taken place in the country or setting where the study was performed.

A large European study involving 38 centers and 5,871 children with diagnosed CD confirmed that there had been an overall mean increase in age at diagnosis from 2 years in 1975 to 4 years in 1990 (39), with Sweden being the exception (40). The changing CD pattern has been described as occurring at different times within and without different countries. In the Naples region of Italy, for example, no change in age at diagnosis was observed between 1973 and 1986 (41). In some other parts of Italy, however, an increase in age was observed (39), with classical forms of CD decreasing in the late 1990s and early 2000s and silent forms significantly increasing, resulting in an increased age at diagnosis as well (42). Similarly, no changes in the overall clinical makeup of CD could be found in the Netherlands between 1975 and 1994 (43) but an increased awareness and recognition of childhood CD did ultimately change the clinical presentation during the years 1993–2000 (44). In the UK, considerable changes were reported mostly due to targeted serum antibody screening. A decrease in the proportion of patients presenting with gastrointestinal manifestations and a rise in the number of patients without symptoms was also observed (45). This change was still occurring in the UK during the years 2005–2011, with over 50% of children diagnosed with CD exhibiting few or no symptoms (46). This same type of changing clinical pattern has been observed in Greece (47), North America (48–50), India (51, 52), Estonia (53), Turkey (54), and Sweden (although, in this case, only after mid-1990s when the Swedish epidemic occurring in young infant from 1985 to 1995 had ended) (40, 55). In Finland, between 1975 and 1990, large differences in CD incidence rates were found when comparing Helsinki to Tampere (39), since an awareness of clinically silent CD was not acknowledged by primary health care professionals in the capital of Finland. Later, however, in Helsinki, the number of patients with CD was found to increase six-fold between 2000 and 2005, with mean age at diagnosis increasing to 7.2 years (56). When comparing the two neighboring countries of Finland and Sweden based on CD's clinical features from 1985 to 1989, the differences found were remarkable: in Sweden, classical clinical symptoms and a diagnosis before the age of two was most commonly observed, whereas, in Finland, symptoms varied much more greatly, and diagnosis was often made after the age of 8 (57). However, the presentation of CD in Finnish children seems to no longer be shifting, with a plateau having been reached in recent years (58).

These observed changes in clinical presentation had inevitable effects on the epidemiological outcomes of CD and were, in terms of timeframe and magnitude, not similar between and among the different countries. It is thus difficult to compare different CD incidences from different publications and countries. Overall, a change in the pattern of symptom presentation for childhood CD that is in line with early observations has been noted in the textbooks as well as in CD guidelines worldwide (7, 59–61).

In Finland, during the 1970s, there was a growing awareness of the aforementioned changing CD pattern, as shown in Figure 1, which influenced health care professionals' case-finding via screening. In fact, this awareness seemed to be the major factor involved in the changing epidemiology and increasing incident rate observed in older children. However, it should be noticed that changing epidemiology is also a result of whether disease-specific autoantibody tools are used for case-finding in the primary healthcare field instead of only in secondary or tertiary referral centers. Today, children with CD are not often so symptomatic that they would need to be admitted to pediatric gastroenterology centers. Furthermore, case-finding using autoantibody screening and a high index of disease suspicion is in our experience a prominent diagnosis method in primary care. Finland also experienced a decrease in the yearly incidence rate during the economic crisis of the early 1990s, during which the city of Tampere health authority forbade primary care doctors to use reticulin or endomysial autoantibodies as case-finding tools, suggesting instead the cheaper but outdated crude gliadin serum antibody test. The authority intervention resulted within a 2-year timeframe in a three-time decrease of biopsy-verified diagnoses at the referral center.

The current major question in pediatrics asks what has caused the described change in the clinical presentation of CD over time. When addressing the changing epidemiology of childhood CD in 1992 (39), we suggested the following environmental factors to play a role: genetic background of the population, infantile gastroenteritis/other infections, unmodified/adapted cow's milk formulas, breastfeeding, age at gluten introduction, quantity of gluten, quality of cereals, and quality of wheat gluten (62). These environmental factors will be discussed here in more detail. The present review is not extended to include human leukocyte antigens (HLA), non-HLA genes, or other genetic aspects in populations with low prevalence rates of CD, such as those comprised of black Africans or Japanese individuals, but did include environmental factors in populations with similar HLA DQ2 and DQ8 background. Environmental factors have an evident impact on intestinal epithelial cells, either through direct interaction or through microbiota. This review does not address any potential epigenetic mechanisms, such as histone modifications, DNA methylation, or microbial RNA methylation, as indicators of the changing pattern in CD epidemiology over time.

## Envionmental Factors Behind Changing Patterns of Celiac Disease

### Infant Feeding Patterns

#### Breastfeeding

As early as 1953, Andersen and di Sant'Agnese found that breastfeeding for a duration of more than 2 months delayed the onset of diarrhea in patients with CD (63). Moreover, two case-control studies performed in Italy indicated breastfeeding protected patients against CD (64, 65). When evaluating whether the duration of breastfeeding influenced age at diagnosis, a mean increase in breastfeeding from 2.5 months in children born from 1961 to 1965 to 3.9 months in children born from 1976 to 1980 was observed. Furthermore, a significant correlation was found between age at diagnosis and duration of breastfeeding in a study of 45 children diagnosed with CD before the age of 6 years, with breastfeeding found to delay the onset of symptoms (23). Thus, prolonged breastfeeding seemed to prevent the development of CD but did not protect patients against the disease, since it manifest at a later age. Overall, the median age at diagnosis was 8.8 years in Tampere, Finland during the early 1980s (39). In the neighboring country of Sweden, at the time of their CD epidemic in 1985–1995 (described below) and a national trend in short breastfeeding duration (40), the median age at childhood diagnosis was 1.3 years (57). Table 1 shows the percentages of Finnish, Italian, and Swedish children breastfed at the ages of 6, 9, and 12 months during a time in which the diagnoses of children under two displayed values of 7, 40, and 74%, respectively (57, 62). At the same time, in the UK and Ireland, it was observed that breastfed babies presented with CD later than their bottle-fed counterparts (22, 24). In 2002, Ivarsson et al. revealed the protective effect of breastfeeding against CD (66).

**Table 1:** Amounts of ingested gluten-containing cereal proteins (g per protein per kg body weight per day) and percentage of breastfeeding of healthy Finnish (Tampere), Swedish (Gothenburg) and Italian (Naples) infants at 6, 9, and 12 months of age in 1990 at the time of the Swedish epidemic [adapted from Ascher et al. (57) and Mäki et al. (62)].

	***n***	**Wheat**		**Rye**		**Barley**		**Oats**		
		**Mean**	**SD**	**Mean**	**SD**	**Mean**	**SD**	**Mean**	**SD**	**Breast-feeding %**
**6 months**										
Tampere	73	0.06^a^	0.06	0.02	0.03	0.01	0.02	0.20	0.23	71
Naples	50	0.44^a^	0.24							10
**9 months**										
Tampere	60	0.14^a^	0.09	0.08	0.10	0.03^a^	0.04	0.25^a^	0.17	47
Gothenburg	20	0.48^a^	0.21	0.04	0.05	0.01^a^	0.01	0.16^a^	0.14	20
Naples	58	0.6^a^	0.23							(1 child)
**12 months**										
Tampere	47	0.20^a^	0.13	0.12	0.12	0.03	0.03	0.23	0.15	17
Gothenburg	13	0.42^a^	0.13	0.09	0.05	0.02	0.02	0.20	0.19	0
Naples	58	0.60^a^	0.25							0

a Significant difference between countries.

*In Naples no rye, barley, or oats was consumed*.

As the research has suggested, breastfeeding may prevent the development of CD. Thus, Akobeng et al. conducted a systematic review and meta-analysis of certain observational studies published between 1966 and 2004 (67). The meta-analysis showed that the risk of CD was significantly reduced in infants who were breastfed at the time of gluten introduction (pooled odds ratio of 0.48, 95% CI 0.40–0.59) compared with infants who were not breastfed during this period. A systematic review from 2012 provided a similar picture, with the study concluding that certain studies indicate a protective effect of breastfeeding, while others do not. The meta-analysis reported a lower risk of CD in breastfed infants, further stating, however, that it was unclear whether those breastfed received permanent protection from CD (68).

In 2016, a position paper by the European Society for Pediatric Gastroenterology, Hepatology, and Nutrition (ESPGHAN) stated that breastfeeding has not been shown to reduce the risk of CD in children (69). Indeed, new evidence on breastfeeding not to be protective against CD has emerged from prospective randomized clinical trials (70–72). However, the evaluated studies were not often designed to directly address the effect of breastfeeding on CD. At the time of the changing pattern in CD presentation, prolonged breastfeeding in Finland was associated with a postponed diagnosis of CD but not with its prevention altogether (23). This effect was also observed in Ireland and the UK (22, 24) at the same time, with breastfed infants diagnosed with CD later than their bottle-fed counterparts despite their similar ages at gluten introduction (24). These early reports indicate that breastfeeding could still have contributed to the changing epidemiology of CD (19–24).

#### Age at Gluten Introduction

In our study of the changing pattern observed in childhood CD presentation (Figure 1), no correlation between age at diagnosis and time of gluten introduction was found (23). Meijer et al. presented recent studies as evidence of the effect of gluten introduction timing and the associated risk of CD in young children (73), concluding that it did not influence the risk of CD, as also suggested by the results of large prospective studies. Furthermore, the position paper by ESPGHAN in 2016 recommended (a) to not introduce gluten while the infant is being breastfed as a means of reducing the risk of developing CD (i.e., decreasing the incidence rate of CD); (b) gluten introduction at age 4–6 months, compared to gluten introduction at age >6 months, does not reduce the cumulative incidence rate of CD in children; (c) for children at a high risk of CD, gluten introduction at 6 months of age compared to 12 months of age does not reduce the cumulative incidence rate of CD but instead leads to earlier manifestations; (d) it remains unclear whether gluten introduction at age <3–4 months compared with 4–6 months has an effect on the risk of developing CD; and (e) it remains unclear whether gluten introduction at age <3–4 months compared to gluten introduction at age >6 months has an effect on the risk of developing CD (69).

Even if the current available evidence suggests that age at gluten introduction is not associated with the changing CD epidemiology, this review suggests that it could still have played an important role at the time when chronic diarrhea and failure to thrive disappeared as presenting clinical features of the disease (19–22, 24). It should be noted that in the 1960s and earlier, gluten was introduced to infants at a very early age. From 1960 to 1965 in Ireland, the mean age at gluten introduction for 130 newly diagnosed children was 2.3 months, while, from 1976 to 1981, it was 4.2 months (22). Moreover, in the UK before 1975, the median age at gluten introduction for diagnosed CD patients was 2 months, while, after 1975, it increased to 4 months (24). These changes in infant feeding patterns coincided with the observed change in CD epidemiology.

#### Amount of Gluten and Quality of Cereals

The amount and type of cereals an infant ingests may play an important role in the changing epidemiologic makeup of CD. Since major differences in the incidence rate and presentation of CD in Finland and Sweden were observed, a joint study was conducted to explore potential causes of the phenomena (57). The results from late 1980s showed that the Gothenburg region of Sweden reported 30 times more infants diagnosed with CD before the age of two than Tampere, Finland. At this time in Finland, cereals were implemented gradually into infant diets from the age of 5 months onward, with parents having a choice between different types of gluten-containing cereals (i.e., wheat, rye, barley, oat) or non-gluten-containing cereals (i.e., rice) in the form of gruels or porridge. On the contrary, in Sweden since 1982, there has been a rapid introduction of gluten at the age of 6 months. Table 1 shows the results of a prospective study of dietary intake of gluten-containing cereals in two neighboring countries exhibiting highly significant differences in the amount of gluten ingested and in quality of cereals (57, 62). The Swedish infants at the age of 9 months ingested three times and at the age of 1 year twice the amount of wheat protein compared to their Finnish counterparts (Table 1). The same evaluation was at the same time conducted in Italy in the Naples region. In Italy, weaning food was completely wheat-based. When wheat, rye, barley, and oat proteins are counted at once, the figures of ingested gluten of the Nordic countries converged with those of Italy. The gluten amount ingested during infancy also correspond to the country differences in CD incidence and prevalence at that time (23, 40, 41, 57).

Here it should be noted that “gluten” (i.e., avenin) in oats is not CD-inducing (74–76). Therefore, another important aspect to consider why in the late 1980s Sweden experienced 30 times more infants diagnosed with CD before the age of 2 than Finland, is the amount of oats ingested by the infants: at the age of 9 months, Finnish infants ingested significantly more oat protein than Swedish infants (Table 1).

Low wheat intake has been thought to cause the low incidence rate of CD in the countries of Denmark (77) and Estonia (78). When comparing the prevalence of CD in Denmark vs. Sweden in 1987, it was estimated that, for infants at 8 months of age, the Swedish diet contained 40 times more gliadin than the Danish diet, while, at 12 months of age, it contained four times more. The weaning food in Denmark was based on rye not wheat (77). Rye gluten, i.e., secalin could actually be clearly less disease-inducing than wheat gluten, i.e., gliadin.

More recently, Cresco-Escobar et al. reported that gluten consumption patterns and the amount of gluten consumed between the ages of 11–36 do not influence CD development in children at a genetic risk for the disease (79). Furthermore, a new study from the US was able to link gluten intake volume in 1-year-olds with the future onset of CD (80).

One further potential environmental factor in influencing the presentation of CD might be the changes in the gluten-rich cereal varieties themselves because of wheat breeding. New hybridization techniques have been used to produce new strains of modern wheat where celiac-triggering gluten proteins are expressed to higher levels. These new strain do express even new gluten proteins on which no animal or human safety testing was conducted (81, 82).

#### Cow's Milk as a Contributing Factor to CD

The present review regarded also older publications from the time when changing clinical features were observed and infants were fed unmodified cow's milk. Important modern epidemiology literature using systemic reviews and meta-analyses has not discussed these studies. The third important contributing factor to changing CD epidemiology in infancy, in addition to the introduction of breast milk and gluten, might in fact be the use of cow's milk.

During the 1960s, young children from the Tampere region diagnosed with CD all received homemade, diluted, cooked, “half”- and “two-third”-cow's milk (23, 62, 83). The shift from using diluted cooked cow's milk to humanized, i.e., more breast milk resembling cow's milk-based infant formulas took place in Finland during the year 1972. This, together with the decrease in the amount of ingested gluten as a result of prolonged breastfeeding, could have significantly contributed to the disappearance of the symptomatic forms of CD in young children. In the UK, bottle-fed infants presented earlier with CD when compared to breast-fed infants (24). This was true both prior to 1975 when gluten was introduced at a median age of 2 months as well as after 1975 when gluten was introduced at a median of 4 months. Before the mid 1970s, most artificially-fed Irish infants received formulas containing unmodified cow's milk, with the total protein content and osmolarity of such formulas both reduced at the same time as the decreasing incidence rate of CD was observed. Humanized infant formulas were implemented from 1978 onwards both in the UK and Ireland (22).

Kuitunen et al. reported that malabsorption syndrome caused by cow's milk protein intolerance in patients from 1962 to 1971 was a clear-cut clinical entity; gluten was not the causing factor of the disease (84). Moreover, small-intestinal biopsies obtained from these infants showed that the jejunal mucosa was injured, the patients displayed villous atrophy with crypt hyperplasia that was undistinguishable from that of untreated CD. The development of a so-called flat lesion was observed in approximately half of the patients (84–86). During the 1960's, a high incidence rate of cow's milk intolerance at infancy was found in diagnosed CD. Kuitunen et al. suggested that cow's milk intolerance may “pave the way” to CD (85).

A recent study by Hyytinen et al. did not find evidence that weaning off a diet of extensively hydrolyzed formula (compared to the implementation of cow's milk-based formula) would decrease the risk of CD later in life (87). It is not clear whether the breastfeeding and gluten introduction timelines presented in this study (87) are comparable with those from the previous, aforementioned studies (22–24). Furthermore, it is also currently unclear whether the cow's milk formula used by Hyytinen et al. (87) (80% intact milk protein, Enfamil; Mead Johnson, Chicago, IL, USA, and 20% hydrolyzed milk protein) mimicked the protein content and osmolarity of the homemade, cooked, diluted cow's milk used during the 1960s and early 1970s.

### National Recommendations Causing Celiac Disease (“The Swedish Epidemic”)

Celiac disease (CD) is an example of when a national recommendation can modify a disease's epidemiology. The trend in Europe of disappearing infant CD and the shift of age at diagnosis (19–24, 38) was not observed to be the same among all countries, with Sweden being an exception (40, 57, 77). In 2000, Ivarsson et al. investigated the countrywide epidemic of chronic malabsorptive syndrome occurring during infancy for a 10-year period from 1985 to 1995. The annual incidence rate of CD in children <2 years of age was found to increase four-fold during the epidemic in Sweden (40). Before the epidemic in 1983, recommendations for age at gluten introduction changed from 4 to 6 months of age, and as previously discussed, there was a rapid introduction of a high amount of gluten at the age of 6 months, inducing CD. In 1983, due to national recommendations, the amount of gluten in industrially produced weaning food in Sweden had been doubled on average. The curve presented by Ivarsson et al. for the wheat-rye-barley index based on the estimated average daily consumption of gluten-containing follow-on formulas followed the pattern of the epidemic in this country (40). However, it was observed that oats were not included in this cereal index curve but the estimated average daily consumption of oats was given (40). So, the curve for oats consumption was drawn exhibiting a U-shape, suggesting that the less oats ingested, the higher the incidence rate of infantile CD. The epidemic disappeared when wheat-based gluten content in the follow-on formulas was reduced (40). Table 1 shows the differences in cereal consumption patterns for the ages of 9 and 12 months in Sweden and Finland.

An important message from the Swedish CD epidemic is that authority directives can completely change the overall makeup of a disease over a short time period.

### Infections and the Microbiome

Infantile gastroenteritis and other infections could be potential environmental factors functioning at the small intestine mucosal level and potentially leading to overt CD in children exhibiting normal mucosa (62). Furthermore, the rotavirus has been implicated as potentially triggering the onset of CD (88). A large cohort study of children with HLA risk alleles for CD found that frequent rotavirus infections (and not clinically symptomatic gastroenteritis) predicted a higher risk of CD autoimmunity (89). Furthermore, rotavirus infection increased CD autoimmunity, while vaccination seemed to reduce this risk (90). In fact, rotavirus infection may cause the manifestation of a small intestinal crypt hyperplastic injury with villous atrophy undistinguishable from the effects of gluten in CD, which was observed in both an experimental animal model and a child with rotavirus infection (91, 92). Recently, an experiment performed by Bouziat et al. on a human reassortant reovirus infection in mice was shown to trigger inflammatory responses to dietary antigens (93). In their study also CD patients tended to present with higher incidence rates of anti-reovirus (but not the rotavirus) antibody levels compared to the controls. Bouziat et al. further concluded that their study in humans supported the role of the reovirus, a seemingly innocuous virus, in triggering the development of CD (93). Recently, an association between enterovirus infection and later CD presentation was also reported (94). Furthermore, there has been evidence that, in general, any type of infection could be associated with an increased risk of CD (95, 96).

Since the rotavirus has been implicated as potentially triggering the onset of CD, live oral rotavirus vaccines could conceivably have a similar effect. However, a Finnish study in 2017 suggested that rotavirus vaccination did not increase the risk of CD in rotavirus-vaccinated children compared to those not vaccinated (97). In fact, rotavirus vaccination could even decrease the occurrence of CD in children and adolescents (98).

Tye-Din et al. discussed the complex role of microbiota in CD development (99). They found that factors such as environment, diet, and antibiotics can change the CD microbiome and its interactions with intestinal epithelial cells. The authors concluded that larger trials are warranted when microbiota are studied in at-risk individuals and must be followed-up with for a certain period of time to further understand gene-microbiome interactions in CD development (99). Recently, an observational, nationwide, register-based cohort study of children was performed in Norway and Denmark from 1995 to 2012 to elucidate the results of systemic antibiotic use in regard to CD (100), which indicated that childhood exposure to systemic antibiotics could be a risk factor of CD.

**Figure 3:**
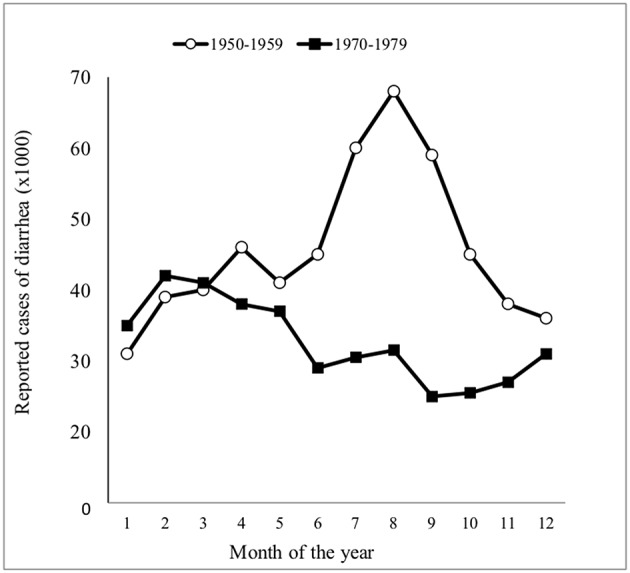
Seasonal distribution of all reported cases of diarrhea in Finland during two 10-year periods [adapted from Mäki et al. (101)].

When examining the environment in which the changing pattern of CD occurred in children (Figure 1), robust epidemiological data supports the influence of gastroenteritis, especially of the bacterial variety rather than the rotavirus-induced disease (101). Figure 3 presents all cases of childhood diarrhea in the country reported to the National Board of Health in Helsinki, Finland over two 10-year periods. Diarrhea cases occurring during the summer, which produced large infant mortality rates, were seen to completely disappear during the 1970s in Finland. Moreover, the shape of the curve was now typical of the rotavirus infection, with the rotavirus also associated with half of all hospitalization cases from the years 1977 to 1978 (102). Additionally, enteropathogenic bacteria accounted for only 10% of all hospitalized cases. These were Yersinia, Campylobacter, certain strains of Salmonella, a few enteropathogenic *Escherichia coli* serotypes (EPEC), and zero Shigellas (102, 103). It should be noted that classical cases of CD in young infants disappeared throughout Finland, even though the rotavirus was seen to greatly induce diarrhea and accounted for 50% of all hospitalization cases at that time (103). EPEC was seen to be most abundant during the summer months of the 1950s and was detected in 24% of all diarrhea cases in children ages 0–3 and in 28% of diarrhea cases for ages 3–6 months (101). Fewer “summer diarrhea” cases were reported during the 1960s; however, they were still quite prevalent and only completed to disappeared during the 1970s (101). Furthermore, the observed rate of hypertonic and hypotonic dehydration was only 3% in 1978, with 97% of all hospitalized children with diarrhea displaying isotonic dehydration (at the time when CD in infants had already disappeared) (101).

There are still some countries reporting the classical forms of CD in young children. For example, in Romania, enteropathogenic bacteria, EPEC, and infestations like giardia are all quite prevalent while very rare in Finland. Indeed, Romania is still currently experiencing several celiac crises, with eight new childhood cases occurring within just 1 year in Bucharest. On the other hand, general autoimmunity and pediatric chronic inflammatory bowel disease occur infrequently in Romania when compared to several other countries.

### Autoimmunity and the Hygiene Theory

The increased prevalence of autoimmune diseases over time in industrialized countries is thought to be caused by a reduction in the incidence rate of infectious diseases, which is called the hygiene hypothesis. Early exposure to certain microorganisms during infancy directs and educates the human immune system. The frequency of infections directly contributes to an increase in autoimmune diseases, while infectious agents suppress autoimmune disorders (104, 105). Dysbiosis of commensal intestinal bacteria also plays an important role in autoimmune diseases, and the mechanisms inducing autoimmunity are a complex combination of various environmental factors. In one study, intestinal microbiome development was examined in children from ages 0–3 in Northern Europe, in which early-onset autoimmune diseases are common, particularly in countries like Finland, while less common in countries like Russia (106). The study then suggested that the immunogenicity of early colonizing symbiotic bacteria could be a potential contributing factor to autoimmunity in humans.

The fact that diarrheal diseases and infections trigger CD and that antibiotic treatment at an early age increases the risk of the disease have already been mentioned in this paper. At the same time, the hygiene theory argues that the presence of microbiota and infections at an early age are essential in suppressing subsequent autoimmunity. CD is, in fact, an autoimmune disease in which, in contrast to other autoimmune diseases, the driving force behind the disease is known: the daily ingestion of gluten (107–110). Furthermore, a shift in the pattern of CD presentation toward older age at diagnosis has been evident (23), with the disease altogether not prevalent in adults prior to the 1970s. Changes in the clinical features of the disease in adults were examined in a study conducted in Scotland from 1960 to 79 (111) and later in Finland, with an increased awareness and use of autoantibody case-finding tools (8) associating with CD's high prevalence in adults (112). In certain population-representative screening studies, an increased prevalence of CD autoimmunity was noted by age: 1.5% in children (113), 2.0% in adults (114), and 2.7 % in the elderly (115). It was further shown that the prevalence of CD (i.e., autoimmunity) at the population level is increasing over time, doubling within the last two decades in Finland (114). Other studies later confirming this finding were conducted in the USA (116, 117). It has also been suggested that, for children, there is an increasing incidence over time (118, 119). Very recently, in fact, an increase in the prevalence in childhood CD was confirmed in Italy, showing a prevalence at the population level of 0.70% in 1994 and 1.58% in 2016 (120, 121).

In Russian Karelia, the population currently appears to be protected against autoimmune diseases. When compared to Finland, type I diabetes in children was six times less prevalent in Russia (122). A similar study comparing the prevalence of CD by screening 1988 schoolchildren from Russian Karelia and 3,654 children from Finland was performed and yielded the same result; CD was five times less frequent in Karelia. This may be associated with a protective environment characterized by inferior prosperity and standard of hygiene in Russian Karelia (123). In Finland, decreased microbial exposure in childhood seems to lead to increasing prevalence of autoimmune diseases and CD. In Russian Karelia, understanding could be enhanced in regard to the environmental factors involved in CD protection, as long as the sharp environmental gradient across the border is still existing. Vatanen et al. (106) have already initiated such studies on the microbiome.

## Incidence Rates and Prevalence

In the 1980s in Tampere, Finland, an increase in the number of childhood CD cases diagnosed annually were reported at the same time as a decrease in incidence rates in both the UK and Ireland were also reported (19–22). As shown in Figure 1, this was due to a greater awareness of the mild form of the disease and atypical cases among school-age children. Additionally, by the late 1970s, the use of the modern tissue autoantibody test of the IgA class began to be used in case-finding, i.e., the R1 reticulin antibody test, later called the endomysial (EMA), and the tissue transglutaminase antibody (TG2-ab) test (12, 15). In 1990, it was reported that a decrease in the incidence rate of CD for children under 2 years of age occurs but that at school age increased significantly (83). This is displayed in Table 2 where all children born in a strict geographical area during 1964–1988 were divided in 5-year birth cohorts and followed up to the age of 16. Table 2 shows the numbers of CD patients diagnosed by the healthcare professional and the 5-year birth cohort incidence rate per year for different ages and the total prevalence during childhood up the age of 16. In the later birth cohort groups infant CD disappeared but the total prevalence of CD appeared stable during childhood (83). In terms of contracting the disease under 2 years of age, the risk for those born from 1964 to 1968 was 5.7-fold compared to those born from 1979 to 1983 or 1984 to 1988. Today, the children from the different birth cohorts are 30–55 years-old, and the current prevalence of CD among the same group could be evaluated, which is probably not the 0.1% shown in Table 2 but rather 10–20 times higher, evidenced by population-based screening studies and the established pattern of increasing prevalence over time (113, 114). In addition, these birth cohorts, when followed-up with for 30–50 years, could further provide insights into this increase in prevalence over time. Thus, it is hypothesized that, based on the prevalence of the increase in population-representative adult materials (114), the two 5-year birth cohorts from the years 1964 to 1968 and 1969 to 1973 present lower total CD prevalence than the three 5-year birth cohorts from the years 1974 to 1988. However, it must be noted once more that, today, new seroconversions can still occur at older ages (115).

**Table 2:** Birth cohort incidence rate of 1,000 live births per year at different ages and total prevalence of celiac disease during 5-year periods from 1964 to 1988 [adapted from reference Mäki and Holm (83)].

**Year of birth (No. of live births)**	**Age of diagnosis (years)**												**Total prevalence (95% confidence interval)**
	** <2**			**2–5**			**6–10**			**11–16**			
	***n***	**Incidence**	***p*^a^**	***n***	**Incidence**	***p*^a^**	***n***	**Incidence**	***p*^a^**	***n***	**Incidence**	***p*^a^**	
1964–1968 (29 056)	13	0.22		3	0.03		2	0.01		11	0.06		1.00 [0.64–1.36]
1969–1973 (24 646)	7	0.14	NS	3	0.03	NS	3	0.02	NS	8	0.05	NS	0.85 [0.49–1.22]
1974–1983 (25 962)	4	0.08	NS	3	0.03	NS	7	0.05	<0.10	10	(0.06)^b^	NS	(0.92)^b^ [0.55–1.29]
1979–1983 (25 466)	2	0.04	<0.01	4	0.04	NS	9	(0.07)^b^	<0.025				
1984–1988 (25 500)	2	(0.04)^b^	<0.01	4	(0.04)^b^	NS							

a Significance as compared to the incidence for birth cohort 1964–1968.

b *Not final incidence and prevalence but at least minimum value*.

The lowest incidence rate per 1,000 live births (0.04) was reported in Estonia during the mid 1970s (78). In 2012, based on a nationwide study of childhood CD, Ress et al. showed a more than 30-fold increase in the incidence rate over a 35-year period in Estonia (53) (Table 3). This was due to an awareness of existing even clinically silent CD and case-finding produced through an autoantibody screening. Denmark and The Netherlands similarly reported very low incidence rates that increased over the years (Table 3), which are speculated to potentially be due to changes in the environment (124, 125). Table 3 also shows the selected incidence rates of CD of several different countries per 1,000 live births every year or per 100,000 people every year.

**Table 3:** Changes in incidence rates of childhood celiac disease over time.

**Author (reference number)**	**Study period**	**Age groups (years)**	**Incidence per 1,000 live births per year**	**Incidence per 100,000 person-years**
**Netherlands**				
George et al. (125) George et al. (126) Steens et al. (44)	1976–1990 1993–1994 1993–2000		0.22 0.54 0.81	
**Denmark**				
Weile et al. (124) Michaelsen et al. (127)	1975–1990 1960–1988	0–18 0–18	0.10 0.09	
Dydensborg et al. (128) Grode et al. (129)	1990–1999 1980–2016	0–18 0–9 (girls)		0.8–1.4 10.80
**Italy**				
Zingone et al. (130) Greco et al. (39) Magazzu et al. (131)	2011–2013 1975–1989 1975–1989	0–19	1 3	27.4
**UK**				
Greco et al. (39) Zingone et al. (132) White et al. (118)	1975–1989 1993–2012 2005–2009		0.44	11.9 11.7
**Sweden**				
Greco et al. (39) Ivarsson et al. (40) Tapsas et al. (133)	1975–1989 1984–1985 1996 1994 2009 2013 2012	0–1.9 0–1.9 0–1.9 0–1.9 2–4.9 5–14.9	2.42	200–240 50 301 10 85 78
**Estonia**				
Ress et al. (53)	1976–1980 1986–1990 1991–1995 2001–2005 2006–2010	0–19		0.10 0.48 1.55 1.59 3.14

Table 4 presents childhood CD prevalence figures, including both the biopsy-supported and serologically detected (celiac autoimmunity) diagnosis types, from various parts of the world. The overall prevalence of CD was observed to be ~1%. However, population-representative screening further revealed that several countries, such as Argentina (135), Finland (113), Hungary (142, 143), Italy (121, 147–149), Spain (154), Sweden (157–160), and Turkey (163), were approaching a childhood prevalence of 1% or more. Meanwhile, countries exhibiting low CD prevalence included, among others, Estonia (138, 139), and Russia (123) (Table 4). The quite low prevalence rate observed for Denmark, even after its increase over the years (128), indicates that CD awareness is low among healthcare professionals and that serum autoantibody case-finding screening in the primary care field is not often performed. Furthermore, a questionnaire-based case-finding was conducted in Denmark in which children exhibiting celiac-related symptoms were invited to participate in a serological test. This evaluation showed that 14 out of 9,967 school-aged children suffer from CD in Denmark. For the same population, an additional 13 patients also had a previous diagnosis of CD; thus, the prevalence rate increased to 0.27% (166). However, this value is still quite low for a country in which screened type 1 diabetes patients are known to often conduct CD as well (10.4%) (167). The high prevalence rate (3%) of CD in Swedish children also can be commented upon (159). In that study in Sweden, the children were born during the epidemic described, experienced high volumes of gluten in their weaning food, and their screening took place when they were 12 years of age. It can be speculated that their high gluten intake at a young age contributed to the 3% prevalence rate. On the other hand, it should also be noted that in the Swedish study the diagnostic criteria for CD were somewhat unconventional, also including symptomatic patients with only increased intraepithelial lymphocyte counts shown in their biopsy results (Marsh class 1). Patients with no symptoms but exhibiting Marsh 1 lesions were again not considered to have CD. Overall, when comparing the incidence and prevalence studies from different countries in which a biopsy was the primary detection method, the poor celiac center pathologist interobserver reproducibility of the small intestinal mucosal injury, i.e., the Marsh classes, must be kept in mind. A normal mucosa graded as Marsh 0 may be Marsh 3 c and vice versa (168, 169).

**Table 4:** Prevalence of childhood celiac disease and celiac autoimmunity in different countries.

**Author (reference number)**	**Study period**	**Prevalence biopsy proven (%)**	**95% Confidence interval**	**Prevalence serology based (%)**
**Australia**				
Chin et al. (134)				0.6
**Argentina**				
Mora et al. (135)	2008–2009	1.30		
**Brasil**				
Pratesi et al. (136)	2003	0.54	0.27–0.57	
**Egypt**				
Abu-Zekry et al. (137)	2001–2004	0.53	0.17–0.89	
**Denmark**				
Dydensborg et al. (128)	1996–1999	0.04		
	2000–2010	0.08		
**Estonia**				
Ress et al. (138)	2004–2005	0.34	0.09–0.88	
Lillemae et al. (139)	1998–1999	0.34	0.09–0.88	0.43
**Finland**				
Mäki et al. (113)	1994	1	0.68–1.33	1.50
**Germany**				
Laass et al. (140)	2003–2006			0.8
**Greece**				
Karagiozoglou-Lampoudi et al. (141)	2009			0.65
**Hungary**				
Korponay-Szabo et al. (142)		1.20		
Korponay-Szabo et al. (143)	2005	1.38	0.94–1.82	
**India**				
Makharia et al. (144)	2008–2009	1.04	0.85–1.25	1.44
**Iran**				
Farahmand et al. (145)	2006–2008	0.5		
Dehghani et al. (146)		0.60		2.00
**Italy**				
Catassi et al. (120)	1994	0.70		1.5
Tomassini et al. (147)	1999–2000	0.94		
Mustalahti et al. (148)	1997–2000			1.1
Bonamico et al. (149)	2007	1.16		
Gatti et al. (121)	2016	1.58		
**Netherlands**				
Csizmadia et al. (150)	1987–1997	0.5		
**Norway**				
Størdal et al. (151)	2008–2011	0.38	0.37–0.39	
**Russia**				
Kondrashova et al. (123)	1997–2001	0.2		
**Saudi Arabia**				
Al–Hussaini et al. (152)	2014–2016			1.5
**Spain**				
Almazán et al. (153)	2009–2012			3
Cilleruelo et al. (154)	2004–2005	1.16	0.54–1.78	
Marine et al. (155)	2004–2007	0.49		
	2006–2007	0.49		
Castaño et al. (156)	1998–1999	0.84		
	after 2.5 years	1.4		
**Sweden**				
Carlsson et al. (157)	1995	2		
Carlsson et al. (158)	2000	1		0.7
Myleus et al. (159)	2005–2006	2.9	2.5–3.3	
Ivarsson et al. (160)	2005–2006	2.90		
	2009–2010	2.20		
**Tunisia**				
Hariz et al. (161)	2003–2005	0.45		0.64
Hariz et al. (162)	2011	0.24		
**Turkey**				
Demirceken et al. (163)		0.9		
Dalgic et al. (164)	2006–2008	0.47	0.38–0.57	1.74
**United Kindom**				
Mustalahti et al. (148)	2000			0.9
**USA**				
Fasano et al. (165)	1996–2001	0.31	0.09–0.80	

## Conclusions

The observed changes in childhood CD presentation and epidemiology have been remarkable during the past 50 years, with overall clinical childhood CD presenting as a malabsorption syndrome transforming into a milder form, resulting in an upward shift of age at diagnosis. The incidence rates and prevalence of childhood CD have been constantly increasing due to case-finding screening strategy. The most important factor in the increasing rates of CD seemed to be an awareness of healthcare professionals of this changing pattern, the existence of which is quite evidence-based. This change took place at different times in different countries, resulting in several global differences. Today, the best case-finding biomarkers for the screening of undiagnosed CD are gluten-dependent serum autoantibody tests, and it is recommended that these tools be used in the primary care field. There is also evidence of an increase in CD prevalence in children over time that is similar to that observed in adults. The main question involved in this change in prevalence asks for the reasons behind these observed clinical changes. This study thus discusses changing environmental factors, such as breastfeeding, gluten, and cow's milk ingestion, and infections as causes for the observed changes. To prospectively readdress these questions using modern epidemiological tools, it is suggested that studies be conducted in countries in which the classic presentation of CD in young children, including diarrhea and malabsorption syndrome, still exists. Overall, an oral tolerance toward gluten could still exist in certain settings, and the environmental factors involved in infantile CD and increasing prevalence of autoimmunity at older ages could still be further investigated by future studies.

## Author Contributions

All authors listed have made a substantial, direct, and intellectual contribution to the work, and approved it for publication.

### Conflict of Interest Statement

The authors declare that the research was conducted in the absence of any commercial or financial relationships that could be construed as a potential conflict of interest.
